# Structure evolution during deposition and thermal annealing of amorphous carbon ultrathin films investigated by molecular dynamics simulations

**DOI:** 10.1038/s41598-020-64625-w

**Published:** 2020-05-15

**Authors:** Shengxi Wang, Kyriakos Komvopoulos

**Affiliations:** Department of Mechanical Engineering, University of California, Berkeley, CA 94720 USA

**Keywords:** Materials science, Physics

## Abstract

The evolution of the structure of amorphous carbon (*a*-C) films during deposition and thermal annealing is of significant interest from both the materials science and application perspectives. However, despite the voluminous literature of studies dealing with the deposition and physical properties of *a*-C films, basic understanding of the structure evolution due to phase change during film growth and heating is fairly sparse and empirical, presumably due to the lack of high-resolution instruments that can probe structural changes at the atomic and molecular levels in real time. Molecular dynamics (MD) is a powerful computational method for studying atomic/molecular-scale movement and interactions. Thus, the objective of this study was to perform MD simulations that provide insight into changes in the structure of ultrathin *a*-C films during deposition and annealing. Simulation results reveal a multi-layer film structure, even for *a*-C films as thin as ~20 Å, the existence of a deposition energy that yields *a*-C films with the highest *sp*^3^ content, the transient and steady-state stages of the structure evolution during annealing at different temperatures, and the changes in the hybridization state (mainly in the bulk layer) encountered during annealing at elevated temperatures. The MD results of this study are of particular importance to applications where the deposition conditions and operation temperature affect the structure and, in turn, the physical properties of ultrathin *a*-C films used as protective overcoats.

## Introduction

Amorphous carbon (*a*-C) is a solid consisting of multiple types of bonding configurations that lack long-range ordered structures and may exhibit deviations in interatomic distances and bond angles due to the presence of a high concentration of dangling bonds^1^. In principle, carbon atoms can form three different bonding configurations, i.e., *sp*^3^, *sp*^2^, and *sp*^1^ atomic carbon hybridizations^2^. Thus, most *a*-C films contain graphite-like (*sp*^2^) and diamond-like (*sp*^3^) carbon crystals. Various studies have shown that the physical properties of *a*-C films, such as hardness, friction, and thermal stability, are mainly determined by the fraction of *sp*^3^ bonding^3–10^.

The development of thin-film deposition techniques during the late 20th century accelerated the use of carbon films in applications requiring protective coatings with excellent tribomechanical properties and coatings with exceptional optical and electrical properties^11,12^. For example, in contemporary hard-disk drives (HDDs) an ultrathin (~20 Å thick) *a*-C film is used to protect the magnetic head and the hard disk against corrosion and surface damage caused by intermittent asperity contact that may lead to mechanical wear and seizure. In addition to outstanding tribomechanical properties, the *a*-C films may be required to exhibit good thermal stability if the device operates at an elevated temperature. An example is heat-assisted magnetic recording (HAMR), a contemporary data storage technology that promises to provide extremely high areal storage densities, in which intensive laser heating is applied to both the head and disk media to enable data to be written in the magnetic medium of the hard disk; thus, the thermal stability of the protective *a*-C film is vital to the functionality and longevity of these HDDs that operate under intense thermal conditions^13,14^. Earlier studies were focused on the investigation of the structure of *a*-C films deposited under various process conditions (e.g., incidence angle, substrate bias voltage and duty cycle, and deposition time) using high-resolution transmission electron microscopy and electron energy loss spectroscopy^15–19^. These studies have revealed a strong dependence of deposition parameters on *a*-C film structure and the deposition conditions for synthesizing *a*-C films with a high *sp*^3^ content. The thermal stability of ultrathin *a*-C films deposited on various substrates has also been assessed in the light of annealing experiments^6,20^.

Despite important insight into the growth and thermal stability of ultrathin *a*-C films derived from aforementioned investigations, knowledge of the structure evolution of these ultrathin films during deposition and thermal annealing is mostly empirical, presumably due to the lack of high-resolution instruments for real-time probing of structural changes at the atomic and molecular levels. Molecular dynamics (MD) is a powerful computational method that promises to bridge the knowledge gap of atomic-scale structural changes instigated in nanometer-thick films during deposition and heating. MD simulations of carbon deposition on diamond performed with the Tersoff or adaptive intermolecular reactive empirical bond-order potential have elucidated the effect of deposition parameters on the *a*-C film structure^21–23^. Graphitization of *a*-C films due to thermal annealing or ion bombardment has also been investigated in MD studies that used the modified Brenner and Tersoff potentials to model atomic interaction^24–26^. The former studies have provided important information about the growth and graphitization of ultrathin *a*-C films, which is difficult, if not impossible, to obtain experimentally. The multi-layer structure of *a*-C films synthesized by energetic deposition processes, such as filtered cathodic vacuum arc^17^, makes the experimental study of structural changes in these films even more challenging, especially for nanometer-thick films.

The evolution of the structure of *a*-C films during thermal annealing strongly influences the continuity, permeability, and nanomechanical/tribological properties of these films. However, the majority of previous MD studies only considered spatial changes in the multi-layer structure of ultrathin *a*-C films before and after annealing at different temperatures, lacking critical time-dependent information about spatial changes in film structure. Knowledge of time-dependent changes in *a*-C film structure during deposition and thermal annealing is critical to the fundamental understanding of the film-growth process and the development of thermally-induced damaging processes, such as graphitization and oxidation. Consequently, the objective of this investigation was to perform a comprehensive MD analysis of ultrathin *a*-C film deposition on silicon followed by thermal annealing, using the Tersoff potential to model carbon-carbon, silicon-silicon, and carbon-silicon atomic interactions. The analysis comprised two simulation phases. First, carbon atom deposition on a silicon substrate was simulated and the evolution of the *a*-C film structure under deposition conditions of various atom carbon kinetic energies was investigated. Second, thermal annealing of *a*-C films with the highest *sp*^3^ content was simulated at various temperatures to illuminate transient changes in the film structure. The MD simulations of this study confirm the multi-layer structure of ultrathin *a*-C films and reveal spatiotemporal variations in the film structure at different annealing temperatures, providing insight into the structural stability of *a*-C films under application conditions where thermal effects are significant.

## Analytical Methodology

### Interatomic potential function

In all the MD analyses of multi-atom systems where interatomic forces are described by a potential energy function, atomic movement is determined by solving a set of differential equations of motion. Commonly used interatomic potential energy functions, such as the one-, two-, and three-body potentials, provide a description of the atom positions. However, these interatomic potentials may fail to describe the different bonding states of atoms, such as carbon and silicon. This inadequacy can be remedied by a bond-order potential that couples a two-body potential with a multi-atom correlation^27^. Using the so-called Tersoff potential, the total energy *E* of an atomic system can be expressed as

1 $$E=\,\sum _{i}{E}_{i}=\frac{1}{2}\sum _{i\ne j}{V}_{ij}\,$$

2 $${V}_{ij}={f}_{C}({r}_{ij})[\,{f}_{R}({r}_{ij})+{b}_{ij}{f}_{A}({r}_{ij})]$$

where *E*_*i*_ is the potential energy of atom *i*, *V*_*ij*_ is the bonding energy between atoms *i* and *j*, *r*_*ij*_ is the distance between atoms *i* and *j*, *f*_*C*_ is a smooth cutoff function used to generate a gradual energy decrease to zero with increasing atomic distance, *f*_*R*_ and *f*_*A*_ are repulsive and attractive two-body potentials, respectively, and *b*_*ij*_ is termed the bond order and represents the bond strength in the local environment, i.e., the bond strength of atoms having more neighboring atoms is characterized by a lower potential energy. The potential parameters are obtained by theoretical and experimental fits of the cohesive energy of element configurations. The first two elements to be fit with the Tersoff potential are carbon and silicon^27,28^. More details about the Tersoff potential can be found elsewhere^29,30^.

### Simulation procedure

**Figure 1:**
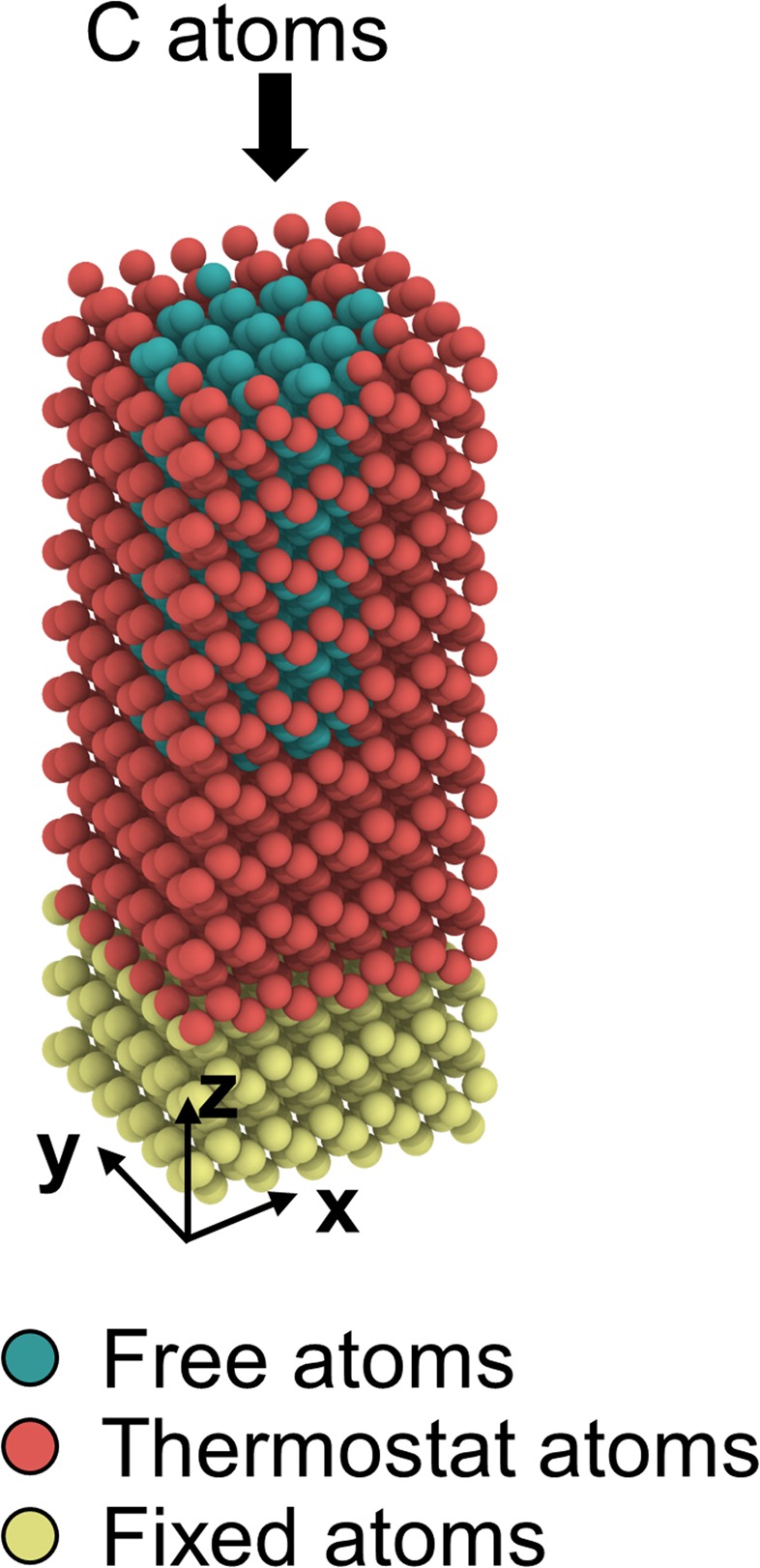
The MD model of the crystalline Si(100) substrate. The yellow atoms at the bottom of the model are fixed to simulate a semi-infinite bulk substrate. The red atoms comprise a Berendsen heat bath that is used to control the temperature of the whole model. The green atoms that are fully surrounded by the thermostat atoms can freely interact with incoming carbon atoms.

As mentioned earlier, the MD simulations of the present study comprise two phases: (1) *a*-C film growth on a Si(100) substrate for various carbon atom kinetic energies and (2) thermal annealing at various temperatures. Film growth was simulated on a 23 × 23 × 59.7 Å silicon substrate consisting of 1620 atoms (Fig. 1). To model an infinitely large deposition area, periodic boundary conditions were applied to the lateral sides of the substrate. The silicon substrate atoms are classified in three categories, shown by different colors in Fig. 1. To simulate a semi-infinite substrate, the two lattice layers at the bottom of the MD model consisting of yellow atoms were fixed. The hollow prism made of red atoms is a Berendsen heat bath that controls the temperature during the simulation process^31^. The green atoms inside the hollow prism of red silicon atoms are free to interact with the incident carbon atoms. This model dissipates the internal energy rise caused by incident carbon atoms and maintains the substrate temperature at the desired level.

Before initiating carbon atom deposition, the silicon substrate was allowed to relax so that to minimize both the energy and the internal stress in the system. This was accomplished by increasing the temperature from 300 to 800 K, maintaining the temperature at 800 K for 5 ps, and finally cooling to 300 K. Thermal relaxation prevented the development of high internal stresses due to the effect of surface tension. After the relaxation of the silicon substrate, 2000 carbon atoms were randomly introduced one at a time from a height of 57 Å above the substrate surface and with velocity normal to the substrate surface. Using a time interval of 2 ps between sequential carbon atom injections allowed the system to relax and reach equilibrium via the Berendsen heat bath using a damping constant of 100 fs. Considering that the thermal spikes generated by bombarding energetic particles of kinetic energy less than 100 eV typically last for less than 0.5 ps^32^, using a time interval of 2 ps was adequate for simulating film growth for a particle kinetic energy of less than 120 eV. In all the deposition simulations, the time step was fixed at 0.5 fs and the heat bath temperature was set at 300 K.

In the thermal annealing simulations, the *a*-C film formed in the deposition phase of the simulation was kept at a constant temperate for 50 ns. The temperature was controlled by the Berendsen heat bath using a damping constant of 100 fs. In all the annealing simulations, the time step was fixed at 0.1 fs. All the MD simulations were performed with the Large-scale Atomic/Molecular Massively Parallel Simulator (LAMMPS) using a node with a 20-core Intel Xeon E5-2670 CPU. To validate the MD model, several simulations were performed to determine the time step and model dimensions that did not affect the results. Representative results from these simulations are shown in Fig. S1 of the Supplementary Information (SI).

## Results and Discussion

### Film growth

**Figure 2:**
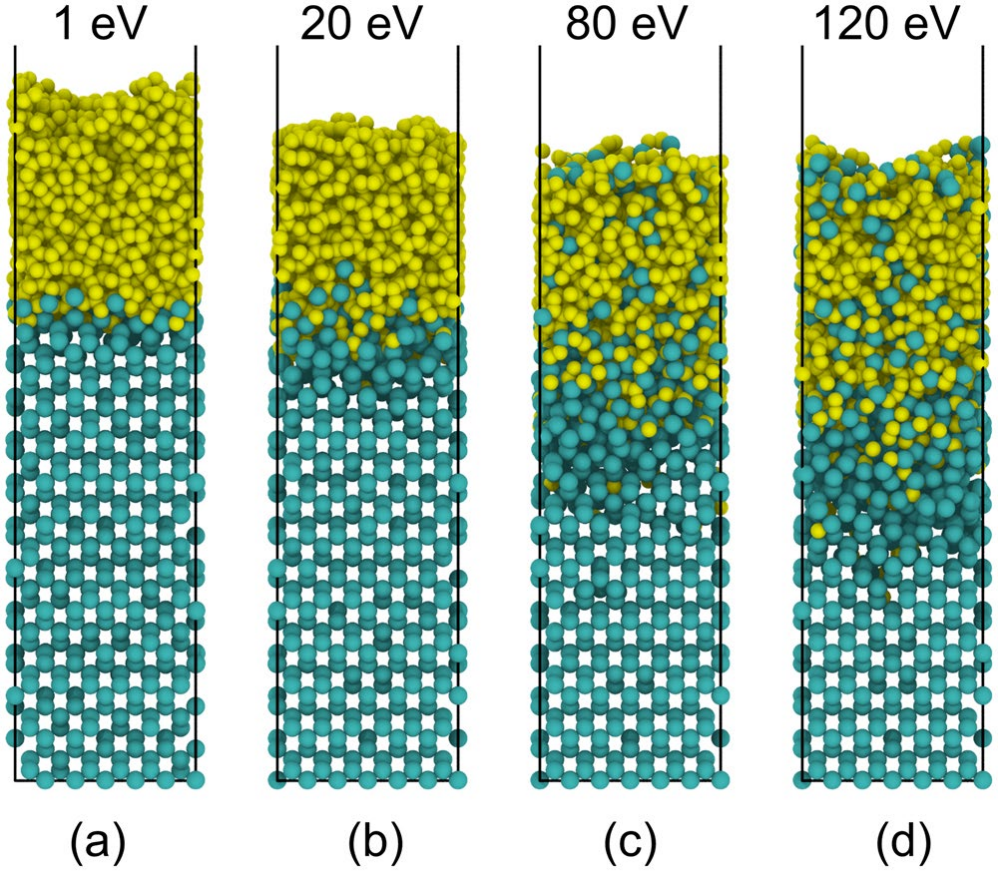
Simulated *a*-C film growth on a crystalline Si(100) substrate for carbon atom deposition energy equal to (**a**) 1, (**b**) 20, (**c**) 80, and (**d**) 120 eV. Silicon and carbon atoms are shown in green and yellow colors, respectively.

Figure 2 shows *a*-C film growth on a silicon substrate for a carbon atom kinetic energy in the range of 1–120 eV. Silicon and carbon atoms are shown in green and yellow colors, respectively. The bombardment of carbon atoms changes the crystalline structure of the silicon substrate, leading to the formation of an amorphous layer at the surface, hereafter referred to as the intermixing layer. The atoms in the intermixing layer comprise various silicon components bonded to neighboring silicon or carbon atoms. The intermixing layer acts as an adhesion layer that bonds the *a*-C film to the silicon substrate. The thicknesses of the intermixing layer and the *a*-C film strongly depend on the kinetic energy of the incident carbon atoms. The carbon atoms with a high kinetic energy can penetrate deeper into the substrate, producing a thicker intermixing layer. The intense bombardment of high energy (i.e., ≥80 eV) carbon atoms also causes a small fraction of silicon atoms to migrate into the *a*-C film.

**Figure 3:**
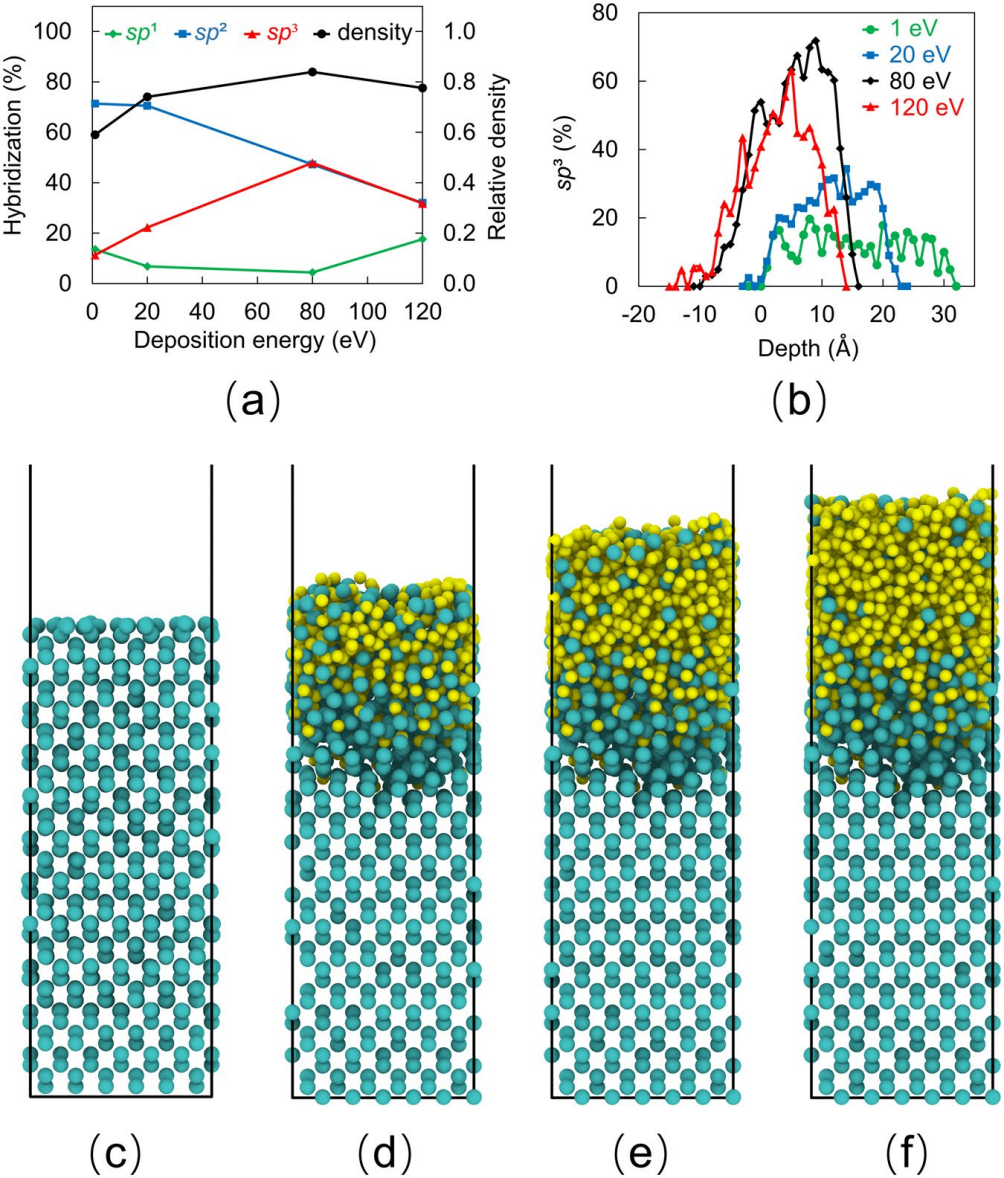
(**a**) Variation of *sp*^1^, *sp*^2^, and *sp*^3^ contents and relative density (normalized by the density of diamond) of *a*-C films with the carbon atom deposition energy, (**b**) *sp*^3^ depth profiles of *a*-C films for carbon atom deposition energy equal to 1, 20, 80, and 120 eV (negative depth values correspond to the silicon substrate), (**c**) silicon substrate after relaxation, (**d**) carbon-silicon intermixing during the initial deposition phase resulting in the formation of an intermixing layer, (**e**) main deposition phase involving the growth of a bulk layer, and (**f**) formation of a surface layer during the end of the deposition process. Silicon and carbon atoms are shown in green and yellow colors, respectively.

The coordination number of carbon atoms affects the structure and properties of the *a*-C film significantly. This number is defined as the number of nearest neighbor atoms and is indicative of the hybridization state, i.e., a coordination number equal to 4, 3, and 2 implies *sp*^3^ (tetrahedral, diamond-like), *sp*^2^ (trigonal, graphite-like), and *sp*^1^ (linear) hybridization, respectively. The coordination number of an atom depends on the cutoff distance of the nearest neighbor atoms. The position of the minimum between the first peak (*r*_1_) and the second peak (*r*_2_) of the radial distribution function is a plausible choice for the cutoff distance. Typical values of *r*_1_ and *r*_2_ for C-C bonds are 1.45 and 2.55 Å, whereas for C-Si bonds *r*_1_ and *r*_2_ are equal to 1.85 and 2.85 Å, respectively. In the present study, the cutoff distance for C-C bonds was set at 1.9 Å, while that for C-Si bonds was set at 2.0 Å, which is close to the 2.1 Å cutoff distance used in previous works^33,34^. Because carbon atoms can form bonds with both carbon and silicon atoms, the *sp*^2^ and *sp*^3^ contents reported here include both C-C and C-Si bonds. Although over- and under-coordinated carbon atoms may co-exist, they represent a very small fraction. Specifically, very few (0.7%) under-coordinated carbon atoms (coordination number = 1) were found at the film surface, which are attributed to the limited atomic bombardment of the outermost atomic plane and the effect of surface relaxation. A very small amount (3.9%) of over-coordinated carbon atoms (coordination number = 5) was found in the as-deposited film structure. Within a very short time from the inception of thermal annealing, all the over-coordinated atoms attained a more stable state corresponding to a coordination number of 2, 3, or 4. Consequently, the effect of under- and over-coordinated carbon atoms on the film structure was secondary. Figure 3(a) shows the *sp*^3^, *sp*^2^, and *sp*^1^ hybridization contents and the relative density of the *a*-C film (defined as the ratio of the film density to that of diamond) versus the deposition energy of carbon atoms. Low deposition energy (e.g., 1 eV) yields a low density and mostly *sp*^2^-hybridized *a*-C film forming by carbon atom physisorption. A graphite-like film structure (~72% *sp*^2^) is produced because the low kinetic energy of impinging carbon atoms is not effective in breaking the thermodynamically stable *sp*^2^ and *sp*^1^ carbon-carbon bonds. The increase of the carbon atom energy enhances carbon-silicon intermixing and *sp*^3^ hybridization in the *a*-C film, resulting in a thicker intermixing layer (Fig. 2) and a thinner, denser, and more *sp*^3^-hybridized film structure (Fig. 3(a)). Energetic incident carbon atoms can break existing carbon-carbon bonds (mainly the relatively weaker *sp*^1^ and *sp*^2^ bonds) and cause resputtering and/or recoil implantation, increasing the film density and promoting *sp*^3^ hybridization. The highest *sp*^3^ content (~48%) and relative density (~0.84) correspond to carbon atom deposition energy equal to 80 eV. Increasing further the deposition energy (e.g., 120 eV) decreases both the *sp*^3^ fraction and the relative density of the film. This is attributed to localized relaxation prompted by thermal spikes and the greater prospect of *sp*^3^ bond breakage and resputtering of deposited carbon material resulting from the intense atomic bombardment. All of the foregoing effects are conducive to graphitization. The similar variations of the *sp*^3^ content and the relative density of the *a*-C film with the increase of deposition energy (Fig. 3(a)) reveal a close dependence of film density on *sp*^3^ content.

It is well-known that *a*-C films synthesized by energetic particle bombardment exhibit a multi-layer structure consisting of intermixing, bulk, and surface layers characterized by significantly different hybridizations^17,35,36^. The bulk layer possesses a much higher *sp*^3^ content than the other two layers. The *sp*^3^ content of the intermixing layer increases gradually from the substrate toward the interface with the bulk layer, while that of the surface layer decreases sharply from the interface with the bulk layer toward the film surface. To examine whether the MD model can simulate the multi-layer structure of *a*-C films, *sp*^3^ depth profiles were computed by averaging the *sp*^3^ content of 1-Å-thick slices through the film thickness. Figure 3(b) shows the effect of carbon deposition energy on the *sp*^3^ depth profile of simulated *a*-C films. All cases reveal a sharply increasing *sp*^3^ content at the film’s interface with the silicon substrate (intermixing layer), a high *sp*^3^ content in the middle region (bulk layer), and a sharply decreasing *sp*^3^ content in the region adjacent to the film surface (surface layer). For very low deposition energy of 1 eV, the *sp*^3^ depth profile is fairly flat, the *sp*^3^ content is below 20%, and the intermixing layer is very thin, indicating the formation of a graphite-like *a*-C film that is slightly integrated with the substrate. Increasing the deposition energy to 80 eV not only enhances carbon-silicon intermixing, which is critical to the bond strength of the film to the substrate, but also decreases the film thickness and increases significantly the *sp*^3^ content in the bulk layer, which controls the overall properties of the *a*-C film. However, the *sp*^3^ content in the bulk layer decreases with the increase of the deposition energy to 120 eV, indicating the existence of an energy threshold for achieving the highest *sp*^3^ content, in agreement with the experimental evidence^19^. Interestingly, all simulation cases show the formation of a surface layer with *sp*^3^ content sharply decreasing toward the film surface, also in agreement with the results of a previous experimental study^15^. The formation of a graphite-like surface layer is attributed to less atomic bombardment than the bulk layer (because it forms at the end of the deposition) and surface relaxation resulting in a minimum energy state. During surface relaxation, the carbon atoms adjacent to the film surface migrate to lower energy sites where it is easier for them to become *sp*^2^ hybridized and the decrease of the intrinsic compressive stress promotes *sp*^3^→*sp*^2^ rehybridization.

The above simulation results and analysis suggest a film-growth process characterized by the sequential main phases depicted in Fig. 3(c–f). An animation of the film-growth process can be seen in Video A of the SI. During the initial deposition phase, carbon atom physisorption and/or implantation are the dominant physical mechanisms, depending on the kinetic energy of the carbon atoms, which control the formation of an ultrathin carbon layer on top of the intermixing layer. Newly arriving carbon atoms collide with previously deposited carbon atoms, knocking them deeper into the substrate (recoil implantation) and contributing to the formation of the intermixing layer (Fig. 3(d)). An animation of recoil implantation where a carbon atom at the surface of the silicon substrate is knocked to a deeper location by an impinging energetic carbon atom can be seen in Video B of the SI. This deposition phase ends when the substrate becomes fully saturated with carbon atoms and carbon-silicon atom interaction diminishes. Next, the incident carbon atoms impinging onto the formed carbon atom planes lead to the growth of the bulk layer (Fig. 3(e)). The densification of the bulk layer and the evolution of high compressive stresses that are conducive to *sp*^3^ hybridization and *sp*^2^→*sp*^3^ rehybridization are encountered under deposition conditions of energetic atom bombardment (e.g., deposition energy of 80 eV). In the final deposition phase, the resputtering of weakly bonded carbon atoms decreases and the development of high compressive stresses for *sp*^3^ hybridization diminishes due to less atomic bombardment of the topmost carbon planes. These effects together with surface relaxation are responsible for the formation of a surface layer rich in *sp*^2^ hybridization (Fig. 3(f)).

Table 1 shows the effect of the deposition energy of carbon atoms on the thickness of the intermixing, bulk, and surface layers. The boundaries of these layers were determined by computing the number of carbon atoms *N*_*c*_ in each 1-Å-thick slice. The *N*_*c*_ of the surface layer showed a sharp decrease, whereas that of the bulk layer remained fairly constant. The thickness of each layer was consequently determined from its known boundaries with other layers. For low deposition energy (e.g., ≤20 eV), penetration of the crystalline silicon structure by impinging carbon atoms is minimal; thus, the thickness of the intermixing layer is very small (2–3 Å). The increase of deposition energy (e.g., ≥80 eV) enhances carbon atom penetration, direct implantation, and recoil implantation in the silicon substrate, producing a significantly thicker intermixing layer. Indeed, for deposition energy equal to 80 and 120 eV, the thickness of the intermixing layer is 12 and 15 Å, respectively, corresponding to about 2.2 and 2.8 times the silicon lattice constant (5.431 Å). In contrast to the intermixing layer thickness, the bulk layer thickness decreases with increasing deposition energy because carbon atoms with a higher kinetic energy are implanted into the silicon substrate, contributing to the formation of a thicker intermixing layer than the growth of a thicker bulk layer. Nevertheless, the effect of carbon atom deposition energy on the surface layer thickness is secondary. This is attributed to less pronounced atomic bombardment of the surface layer in the last deposition phase and the effect of surface relaxation, as explained above.

**Table 1:** Thickness of intermixing, bulk, and surface layers comprising the multi-layer structure of *a*-C films versus carbon atom deposition energy.

Deposition energy (eV)	Thickness (Å)		
	Intermixing layer	Bulk layer	Surface layer
1	2	33	6
20	3	22	3
80	12	13	4
120	15	11	3

### Thermal annealing

MD simulations of thermal annealing were performed for the *a*-C film with the highest (48%) *sp*^3^ content, corresponding to carbon atom deposition energy of 80 eV (Fig. 3(b)). To examine the thermal stability of this film, simulations of thermal annealing were performed for a temperature between 150 and 450 °C. As stated earlier, the thermal stability of ultrathin *a*-C films is critical to the performance of devices operating at elevated temperatures, such as the HDDs designed for HAMR. Thus, the selected range of annealing temperatures is typical of the temperature range of *a*-C films used as protective overcoats of HAMR media.

**Figure 4:**
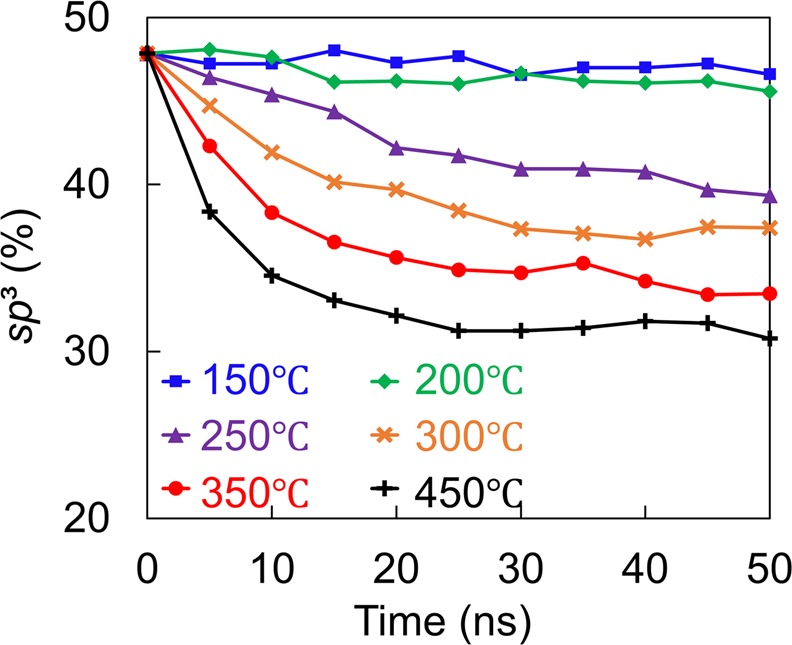
Variation of the overall *sp*^3^ content of *a*-C film with 48% *sp*^3^ content versus annealing time and temperature in the range of 150–450 °C.

Figure 4 shows the overall *sp*^3^ film content (computed as the average of the *sp*^3^ contents of the intermixing, bulk, and surface layers) versus annealing time in the temperature range of 150–450 °C. The film’s structural stability is determined by the minimum temperature that causes the *sp*^3^ fraction to decrease sharply, resulting in *sp*^3^→*sp*^2^ rehybridization. Annealing at 150 and 200 °C decreases the *sp*^3^ fraction by only ~2%, whereas annealing at 250, 300, 350, and 450 °C decreases significantly the *sp*^3^ fraction by 9%, 11%, 15%, and 17%, respectively. These predictions are in agreement with experimental results showing that annealing at 350 °C causes a decrease of the *sp*^3^ content of *a*-C films deposited on various substrates by 4–16%^20^. For annealing at 300, 350, and 450 °C, the *sp*^3^ content reaches a steady state in a very short time, indicating that rehybridization is a very rapid process. The increase of the annealing temperature decreases the time for the *sp*^3^ fraction to reach a steady state. Table 2 shows the effect of annealing temperature on the thickness of the intermixing, bulk, and surface layers. The thickness of the surface layer increases with the temperature at the expense of the bulk layer thickness, while the thickness of the intermixing layer remains constant. The latter is in agreement with electron energy loss spectroscopy results of a previous study^20^ showing that annealing at 350 °C yields minor changes of the intermixing layer thickness of *a*-C films deposited on Au, SiN, NiCr, TaO_x_, and FeCo substrates.

**Table 2:** Thickness of intermixing, bulk, and surface layers comprising the multi-layer structure of *a*-C film with 48% *sp*^3^ overall content obtained after annealing versus annealing temperature.

Annealing temperature (°C)	Thickness (Å)		
	Intermixing layer	Bulk layer	Surface layer
150	12	13	4
200	12	13	4
250	12	13	5
300	12	12	5
350	12	12	6
450	12	12	6

**Figure 5:**
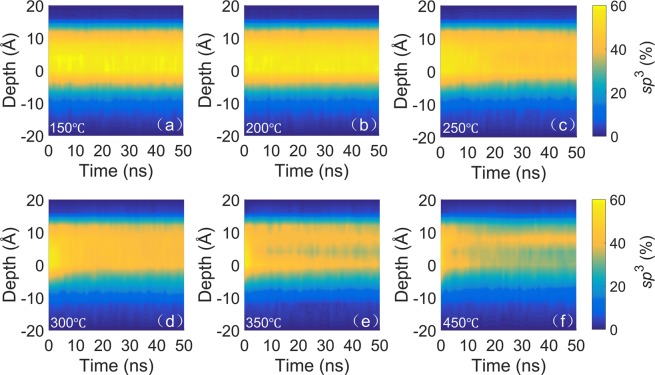
Depth profiles of *sp*³ fraction of *a*-C film with 48% *sp*^3^ content versus annealing time and temperature equal to (**a**) 150 °C, (**b**) 200 °C, (**c**) 250 °C, (**d**) 300 °C, (**e**) 350 °C, and (**f**) 450 °C. Negative depth values correspond to the silicon substrate.

Although Fig. 4 shows the evolution of the *a*-C film structure due to heating, i.e., *sp*^3^→*sp*^2^ rehybridization, it does not provide insight into spatiotemporal changes of the film structure. Thus, to investigate localized changes in the film structure in the course of annealing, the variation of the *sp*^3^ fraction through the film thickness with the annealing time and temperature was examined. As shown in Fig. 5(a,b), the effect of annealing at 150 and 200 °C on the spatial and temporal variation of the *sp*^3^ fraction is practically insignificant, consistent with the invariance of the overall *sp*^3^ content at these temperatures (Fig. 4). However, annealing at 250 °C causes a discernible decrease in the *sp*^3^ content of the bulk layer (Fig. 5(c)), with the effect intensifying as the temperature is further increased (Fig. 5(d–f)). Different from the profound heating effect on the structure of the bulk layer, the effect of annealing on the structure of the intermixing and surface layers is much less pronounced, although a similar decreasing trend of the *sp*^3^ content of these layers is also observed with increasing temperature. For instance, annealing at 250 °C causes the overall *sp*^3^ content to decrease by 9%, with a 12% decrease in *sp*^3^ content sustained by the bulk layer, while the change of the *sp*^3^ content of the intermixing and surface layers is relatively insignificant. Annealing at a temperature above 250 °C intensified the rehybridization process in the bulk layer, which controls the overall *sp*^3^ content of the *a*-C film. Specifically, annealing at 300, 350, and 450 °C resulted in the decrease of the overall *sp*^3^ film content and the bulk layer *sp*^3^ content by 11% and 17%, 15% and 20%, and 17% and 25%, respectively. As with the overall *sp*^3^ content, the *sp*^3^ content at each depth also reached a steady state within a very short annealing time.

**Figure 6:**
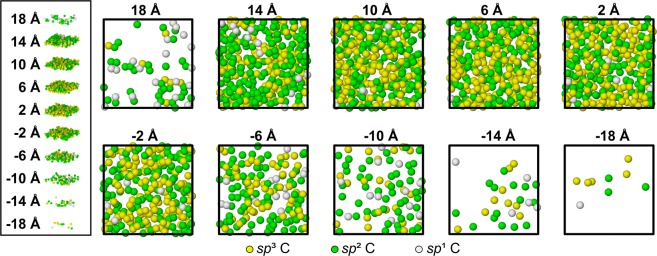
Carbon atoms of different hybridizations in 4-Å-thick horizontal (*x-y*) slices of *a*-C film with 48% *sp*^3^ content. The exploded view on the left shows the depth of each slice. Negative depth values correspond to the silicon substrate. Silicon atoms are not shown for clarity.

Additional insight into the evolution of the film structure was obtained by examining the spatial distribution of carbon atoms in *sp*^1^, *sp*^2^, and *sp*^3^ hybridizations. This was accomplished by slicing the *a*-C film with 48% overall *sp*^3^ content (Table 1) into ten 4-Å-thick horizontal (*x-y*) slices, as shown in Fig. 6. The spatial distributions of carbon atoms with different hybridizations reveal a multi-layer film structure. The first slice (*z* = 18 Å), representing the surface layer of the *a*-C film, contains a few carbon atoms that are mostly *sp*^2^ hybridized. Thus, it may be inferred that the surface layer of the *a*-C film possesses a graphite-like porous structure of relatively low density. The slices corresponding to the bulk layer (2 Å ≤ *z* ≤ 14 Å) contain roughly the same number of carbon atoms, which is significantly higher than that of the surface layer. These slices show a gradual increase in *sp*^3^ hybridization with increasing depth in the range 6 Å ≤ *z* ≤ 14 Å, followed by a slight decrease in *sp*^3^ hybridization at a depth *z* = 2 Å, which is adjacent to the interface of the bulk layer with the intermixing layer. The slices assigned to the intermixing layer (*z* < 0) show a sharp decrease in *sp*^3^ hybridization and carbon atom density with increasing depth. The foregoing variation of the *sp*^3^ content of the surface, bulk, and intermixing layers is consistent with the *sp*^3^ depth profile corresponding to a carbon atom deposition energy equal to 80 eV (Fig. 3(b)) and in qualitative agreement with experimental findings^19^.

**Figure 7:**
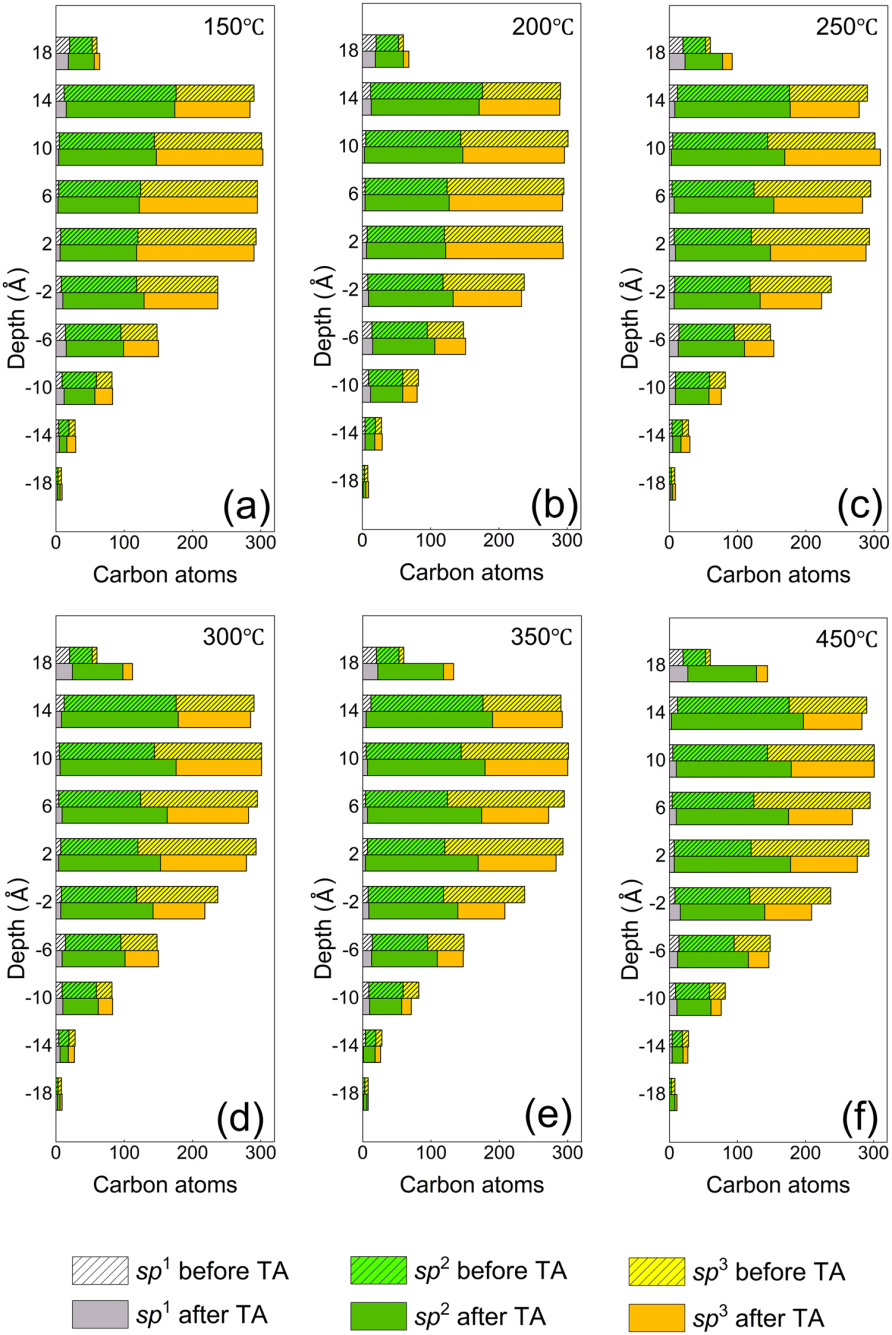
Number of carbon atoms of different hybridizations in 4-Å-thick horizontal (*x-y*) slices of *a*-C film with 48% *sp*^3^ content before and after thermal annealing (TA) at a temperature of (**a**) 150 ^o^C, (**b**) 200 ^o^C, (**c**) 250 ^o^C, (**d**) 300 ^o^C, (**e**) 350 ^o^C, and (**f**) 450 ^o^C.

The data shown in Fig. 6 provide a reference for the annealing simulations. Figure 7 shows the number of carbon atoms in different hybridizations at various depths through the film thickness before and after thermal annealing in the temperature range of 150–450 °C. Figures 7(a,b) show that annealing at 150 and 200 °C, respectively, does not change significantly the carbon atom density and the hybridization state. However, as evidenced from Fig. 7(c–f), annealing at a temperature above 200 °C changes significantly the number of carbon atoms in each slice and also triggers *sp*^3^→*sp*^2^ rehybridization, more notably in the bulk layer (0 ≤ *z* ≤ 13 Å). For instance, while annealing at 250 °C increases the number of carbon atoms in the surface layer (13 Å ≤ *z* ≤ 18 Å) by 29 (46%) and decreases those in the bulk layer (0 Å ≤ *z* ≤ 13 Å) by 32 (2.3%), annealing at 450 °C causes the carbon atoms in the surface layer to sharply increase by 84 (140%) and those in the bulk layer to decrease by 78 (5.5%). Carbon atom rehybridization induced by annealing decreases the *sp*^3^ content of the bulk layer by 9% at 250 °C and 16.7% at 450 °C. Compared to the bulk and surface layers, the intermixing layer exhibits insignificant changes in carbon atom number at all temperatures. The carbon atom changes in the surface layer and the intermixing layer indicate atom migration during annealing, which is attributed to stress relaxation of the bulk layer. The increase of carbon atoms and insignificant hybridization changes in the surface layer indicate the development of a thicker surface layer as a result of annealing at an elevated temperature (i.e., >250 °C). The decrease of the number of carbon atoms and the *sp*^3^ content of the bulk layer due to annealing in the temperature range 250–450 °C suggests the development of a thinner, lower density, and partially graphitized bulk layer. The numbers of *sp*^2^- and *sp*^3^-hybridized carbon atoms in each slice versus annealing temperature shown in Fig. S2 (SI) provide further evidence for the instigation of *sp*^3^→*sp*^2^ rehybridization mainly in the bulk layer.

**Figure 8:**
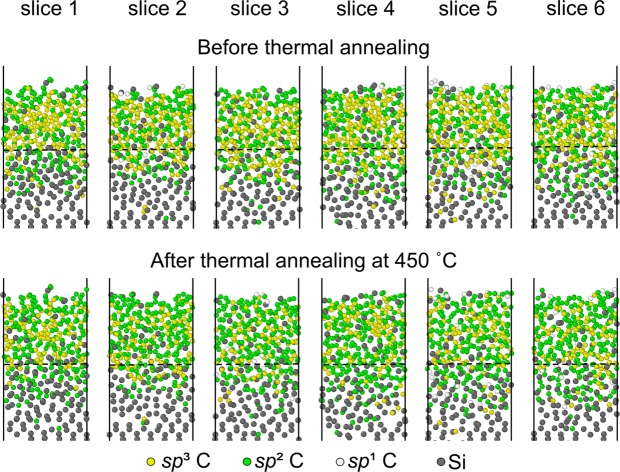
Carbon atoms of different hybridizations in adjacent 4-Å-thick vertical (*y-z*) slices of *a*-C film with 48% *sp*^3^ content before and after annealing at 450 °C. The region below each dashed line is the silicon substrate. The few under-coordinated carbon atoms are shown with the same color as the *sp*^1^ atoms.

In addition to the distributions and hybridizations of carbon atoms in horizontal slices through the film thickness, carbon atom hybridization in 4-Å-thick vertical (*y-z*) slices was also examined to identify any changes in the as-deposited film structure due to annealing at planes perpendicular to the film surface. Figure 8 shows *sp*^1^, *sp*^2^, and *sp*^3^ hybridized carbon and silicon atoms in vertical slices before and after annealing at 450 °C. The simulations reveal two important results. First, only changes in hybridization can be observed after thermal annealing, whereas differences in the number of carbon atoms in each vertical slice are insignificant. In the light of this result and the periodic boundary conditions, it may be inferred that the film possesses a fairly uniform structure. Second, a comparison of carbon atom hybridizations in each slice before and after annealing shows a uniform trend for *sp*^3^→*sp*^2^ rehybridization throughout the film structure, especially in the bulk layer.

In some elevated-temperature applications, the decrease of the *sp*^3^ content of the protective *a*-C film as a result of heating may enhance its chemical reactivity, especially in an oxidizing atmosphere. The evolution of the *a*-C structure in a hot, oxidizing atmosphere could be more complex than that simulated for inert thermal annealing conditions. Thermal annealing in an oxidizing atmosphere may accelerate oxidation and material loss at the film surface, with the thermally excited oxocarbon molecules entering the oxidizing gaseous environment. The reaction of an oxidizing gas with the film in conjunction with the development of mechanical traction due to surface contact may promote or even accelerate carbon oxidation at elevated temperatures, thus degrading the structural integrity of the *a*-C film and, in turn, its protective effectiveness. The detrimental effect of oxidizing gas diffusion on the film’s structural stability may be more pronounced than that of *sp*^3^→*sp*^2^ rehybridization propelled by thermal effects. An MD analysis of ultrathin *a*-C films rich in *sp*^3^ hybridization exposed to an elevated temperature in an oxidizing atmosphere is the objective of a forthcoming publication.

## Conclusions

A comprehensive MD analysis of energetic carbon atom deposition on silicon was performed to elucidate the growth process of ultrathin *a*-C films and identify the structural changes induced by thermal annealing. The MD simulations confirmed the multi-layer structure of *a*-C films synthesized by energetic particle deposition methods. The study of the effect of carbon deposition energy on the film growth characteristics revealed a deposition energy threshold for synthesizing *a*-C films of maximum density and *sp*^3^ hybridization. By increasing the deposition energy toward the deposition energy threshold, the intensifying carbon atom bombardment promoted direct and recoil implantation and the development of high compressive stresses, which are conducive to film/substrate intermixing, preferential resputtering of weakly bonded atoms, *sp*^3^ hybridization, and *sp*^2^→*sp*^3^ rehybridization. However, for deposition energy above the deposition energy threshold, the intense bombardment of the growing film promoted resputtering and, presumably, the development of thermal spikes, which reduced the film density and high compressive stresses, favoring *sp*^2^ hybridization and *sp*^3^→*sp*^2^ rehybridization.

The MD simulations of thermal annealing of the *a*-C film with the highest *sp*^3^ content (48%) in this study demonstrated insignificant structural changes for temperatures up to 200 °C, with progressively intensifying carbon atom migration in the intermixing and surface layers and *sp*^3^→*sp*^2^ rehybridization in the bulk layer with the increase of temperature from 250 to 450 °C. While annealing increased the thickness of the surface layer, it decreased the thickness of the intermixing layer and the density of the bulk layer. An important finding is that *sp*^3^→*sp*^2^ rehybridization at an elevated temperature is a very rapid process. Annealing at a relatively high temperature (e.g., 450 °C) may also have a negative effect on the film’s permeability because it may create potential oxidizing gas diffusion sites.

The results of this study provide new insight into the dependence of the growth and structure of *a*-C films on deposition process conditions and annealing at various temperatures. The knowledge derived from the present MD simulations has direct implications on the physical properties of ultrathin *a*-C films used as protective overcoats of devices operating at elevated temperatures. The good agreement of the simulation trends and results of this study with experimental findings of earlier investigations validate the present MD model and employed analytical methodology. This work provides impetus for using the present MD modeling approach to guide the selection of appropriate film deposition and annealing conditions and to perform parametric studies aimed at tuning the multi-layer structure of *a*-C films according to specific application requirements.

## Supplementary information

Supplementary information.

Supplementary information 2.

Supplementary information 3.
