# Supplementary material for: Structure evolution during deposition and thermal annealing of amorphous carbon ultrathin films investigated by molecular dynamics simulations
Source: Sci Rep. 2020 May 15;10:8089. doi: 10.1038/s41598-020-64625-w (PMC7229151; doi:10.1038/s41598-020-64625-w)
Supplement: Supplementary file 1 — Supplementary information. [file 41598_2020_64625_MOESM1_ESM.docx]

**Structure evolution during deposition and thermal annealing of amorphous carbon ultrathin films investigated by molecular dynamics simulations**

Shengxi Wang & Kyriakos Komvopoulos*

Department of Mechanical Engineering, University of California, Berkeley, CA 94720, USA

**Supplementary Information**

**1. Supplementary Data**

The effects of the substrate size and time step on the simulation results were examined before selecting the final settings of the MD model. To investigate the effect of the substrate dimensions on the simulation results, four substrates with in-plane dimensions 7.68 × 7.68, 15.36 × 15.36, 23.04 × 23.04, and 30.72 × 30.72 Å were used to simulate carbon film growth for an atom kinetic energy of 80 eV. In all four models, the substrate size in the *z*-direction was fixed at 59.74 Å, which is sufficiently large for modeling a half-space substrate, and the time step was set at 0.5 fs. Figure S1(a) shows *sp*^3^ depth profile for each substrate size. The 23.04 × 23.04 and 30.72 × 30.72 Å substrate sizes yield very similar results, while for smaller substrate sizes the *sp*^3^ depth profile exhibits significant variation. It is noted that the total number of carbon atoms introduced into the MD system was adjusted according to the substrate size to yield films of similar thickness. For example, while 2000 carbon atoms were deposited onto the 23.04 × 23.04 Å substrate, only 889 carbon atoms were deposited onto the 15.36 × 15.36 Å substrate, i.e., ~3.8 atoms/Å^2^ in both cases. In view of the results shown in Fig. S1(a), the substrate dimensions in the MD model were selected to be 23.04 × 23.04 × 59.74 Å.

The effect of the time step was examined by performing simulations of *a*-C film growth at an atom kinetic energy of 80 eV on a 23.04 × 23.04 × 59.74 Å substrate for a time step equal to 0.25, 0.5, 1, and 2 fs. Figure S1(b) shows similar *sp*^3^ depth profiles for 0.25 and 0.5 fs time step, whereas the *sp*^3^ depth profile for 1 fs demonstrates more pronounced fluctuations. The simulation with a 2 fs time step resulted in all atoms escaping from the simulation box during post-deposition relaxation because the large time step produced overlapping atoms that encountered very large repulsive forces, leading to model failure. Therefore, a time step of 0.5 fs rather than 0.25 fs was used in the MD simulations to reduce the computational time. The slight difference in the range of the *sp*^3^ depth profiles is due to the random number generator used in the LAMMPS code to generate the incident carbon atoms impinging onto the growing film surface at randomly selected surface positions.

Figures S2(a) and S2(b) show the number of *sp*^2^- and *sp*^3^-hybridized carbon atoms through the film thickness, respectively, after annealing at a temperature in the range of 150–450 ^o^C. The atom numbers represent averages computed from 4-Å-thick horizontal (*x*-*y*) slices through the film thickness. Figure S2(a) shows that the number of *sp*^2^-hybridized carbon atoms in the bulk and surface layers increases dramatically after annealing at 250–450 ^o^C, while the number of *sp*^2^-hybridized carbon atoms in the intermixing layer does not change significantly. The increase of *sp*^2^-hybridized carbon atoms in the surface layer is attributed to atomic migration from the bulk layer toward the surface layer propelled by heating. Figure S2(b) demonstrates an opposite effect for *sp*^3^-hybridized carbon atoms, that is, the increase of the annealing temperature results in a profound decrease of *sp*^3^-hybridized carbon atoms in the bulk layer. The results shown in Fig. S2 provide evidence of the evolution of *sp*^3^⟶*sp*^2^ rehybridization during thermal annealing mainly in the bulk layer of the *a*-C film.


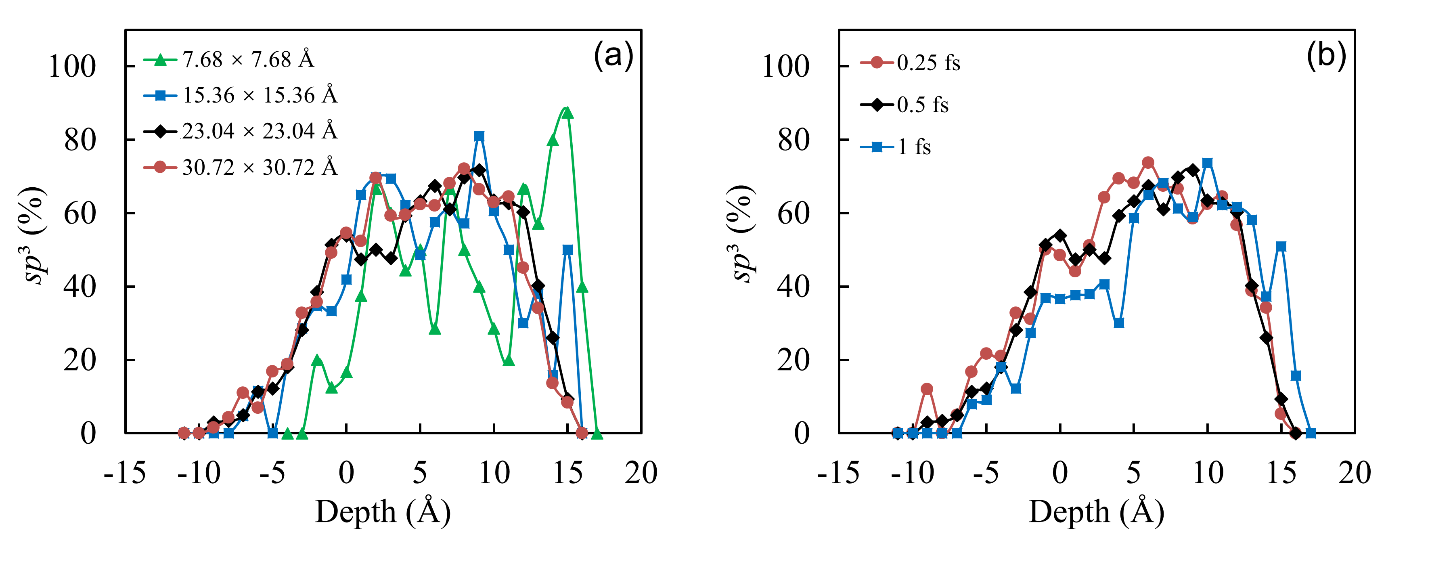
**Figure S1.** Simulated *a*-C film growth on a crystalline Si(100) substrate for a carbon atom deposition energy equal to 80 eV using different models: (a) *sp*^3^ depth profiles for in-plane substrate size equal to 7.68 × 7.68, 15.36 × 15.36, 23.04 × 23.04, and 30.72 × 30.72 Å and 0.5 fs time step and (b) *sp*^3^ depth profiles for in-plane substrate size equal to 23.04 × 23.04Å and time step equal to 0.25, 0.5, and 1 fs.


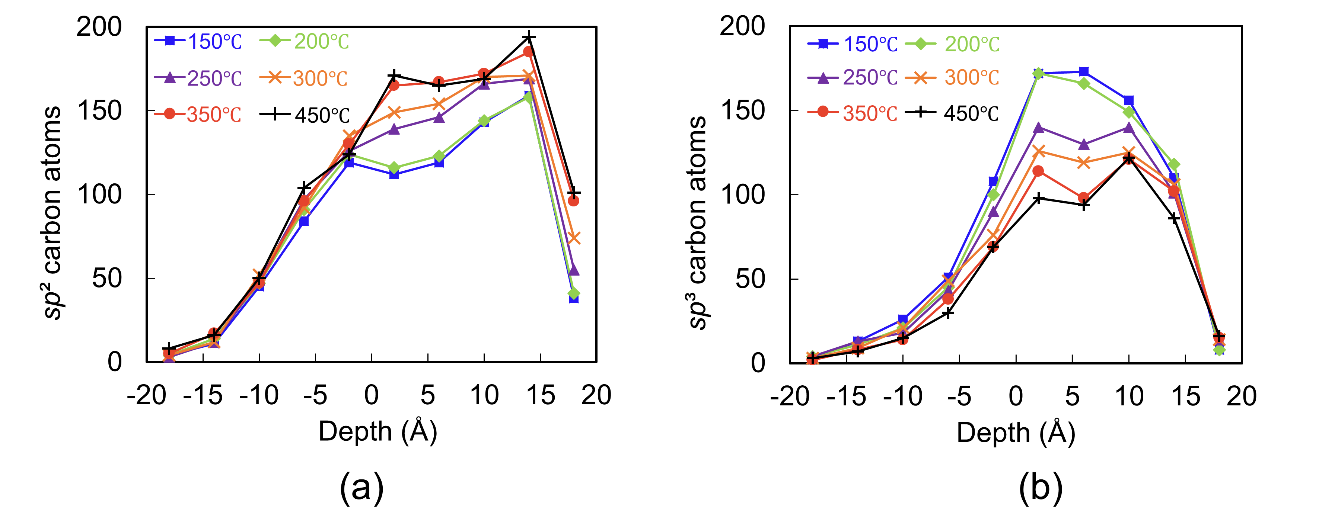
**Figure S2**. Number of carbon atoms with different hybridization states versus depth for *a*-C film with 48% *sp*^3^ overall content after thermal annealing at a temperature in the range of 150–450 ^o^C.

**2. Supplementary Videos**

**Video A.** Animation of the *a*-C film growth process illustrating the sequential formation of the intermixing, bulk, and surface layers.

**Video B.** Animation of recoil implantation. A carbon atom bonded to surface atoms of the silicon substrate is knocked to a deeper location by an impinging energetic carbon atom.
